# Honing the T cell response to HIV: Turning off the noise

**DOI:** 10.1016/j.ebiom.2021.103217

**Published:** 2021-01-28

**Authors:** Nathaniel R. Landau

**Affiliations:** Department of Microbiology, NYU Grossman School of Medicine, NYU Langone Medical Center, New York, NY, USA

HIV infected individuals are treated with a panoply of over 20 antiretroviral medications. Used in combination, the drugs suppress viremia in the large majority of patients but do not eradicate the virus and as a result, patients need to remain on lifelong therapy to keep virus loads in check. In the absence of a means to entirely clear the infection, development of an immunotherapy that fortifies the immune response to the virus might allow for a “functional cure” in which patients could suppress the virus load without therapy. That such an approach could work is supported by the existence of rare elite controllers who, by virtue of a strong antiviral T cell response, hold virus loads in check over several years without therapy. A hurdle in the development of a T cell immunotherapy for HIV is that the immune response to the virus tends to focus on immunodominant viral epitopes that are highly variable and from which the virus easily escapes by mutating in response to selective pressure from antigen-specific CTLs. Further complicating the matter, T cell recognition of viral epitopes is MHC restricted such that individuals respond to different viral epitopes depending on their MHC haplotype, making it difficult to devise a universal vaccine antigen.

In this issue, Garcia-Bates et al. tackle the question of an effective HIV immunotherapy with a dendritic cell (DC) vaccine strategy that elicits CTLs that kill infected cells by targeting ultra-conserved, topologically constrained epitopes from which the virus cannot easily escape [1]. DC vaccines are a promising approach that was applied initially as an immunotherapy to cancer to stimulate T cell responses to tumor antigens but that is equally applicable to HIV [2,3]. DC vaccines rely on the potent ability of these professional antigen presenting cells to present antigens to CD8 T cells and then induce their activation [4]. In this report, Garcia-Bates differentiate infected patient blood monocytes with cytokines to generate type 1 polarized monocyte-derived DCs (MDC1), a DC subset that is particularly good at inducing T cell activation through the release of IL12p70, a Th1 cytokine that promotes cytolytic T cell growth and activation. The MDC1s were then pulsed with pools of synthetic peptides corresponding to HIV sequences and mixed with autologous T cells from HIV-infected donors. The cells used in the study were from donors who were part of a Thai anti-retroviral drug study who had been treated with antiretrovirals during the acute phase of infection, thereby preserving T cell function. The peptide pools were derived by sequence analysis of the virus using two sophisticated computational methods termed “epigraph” [5] and “network” [6]. These methods identify peptides that are predicted to be T cell epitopes that are topologically constrained as a result of their biological function in virus replication. Such epitopes would not be easily mutated and thus not subject to escape and being non-immunodominant, may not have been previously recognized. The analysis identified 14–21mer “afferent” peptides that are efficiently processed and presented to T cells and shorter 9–13mer “efferent” peptides that are directly presented to CTLs without processing. The pools were further chosen to remove immunodominant peptides (turning off immunologic noise), potentially promoting a T cell response that is more effective than that promoted by natural infection. The results showed that the pools were much more effective at priming donor T cells than a more conventional pool of overlapping Gag peptides.

A caveat of the study is the use of donor cells from patients treated in acute infection. In practice, the immunotherapy will need to be used on patients who were not fortunate enough to start treatment in the acute phase. It will be important to show that the approach works using cells from donors treated later in the disease course when it may be more difficult to enhance the T cell response. It will be interesting to see how well the approach works in a clinical trial. It remains unclear as to how controllers manage to keep virus loads in-check. It will be interesting to see whether DCs can be used to enhance T cell responses to the virus in chronic infection where CTLs display tend to be dysfunctional and display signs of exhaustion [7]. The study advances the potential for the development of a therapeutic vaccine for HIV that could be used in the “shock and kill” [8] approaches currently under development to cure HIV infection and highlights the idea that as critical cells of the immune system, DCs can be engineered to good use.

## Declaration of interests

Pr Landau has no conflict of interest to disclose.
